# EPI Light Field Depth Estimation Based on a Directional Relationship Model and Multiviewpoint Attention Mechanism

**DOI:** 10.3390/s22166291

**Published:** 2022-08-21

**Authors:** Ming Gao, Huiping Deng, Sen Xiang, Jin Wu, Zeyang He

**Affiliations:** School of Information Science and Engineering, Wuhan University of Science and Technology, Wuhan 430081, China

**Keywords:** light field images, depth estimation, epipolar plane image, pixel consistency, attention mechanism

## Abstract

Light field (LF) image depth estimation is a critical technique for LF-related applications such as 3D reconstruction, target detection, and tracking. The refocusing property of LF images provide rich information for depth estimations; however, it is still challenging in cases of occlusion regions, edge regions, noise interference, etc. The epipolar plane image (EPI) of LF can effectively deal with the depth estimation because of its characteristics of multidirectionality and pixel consistency—in which the LF depth estimations are converted to calculate the EPI slope. This paper proposed an EPI LF depth estimation algorithm based on a directional relationship model and attention mechanism. Unlike the subaperture LF depth estimation method, the proposed method takes EPIs as input images. Specifically, a directional relationship model was used to extract direction features of the horizontal and vertical EPIs, respectively. Then, a multiviewpoint attention mechanism combining channel attention and spatial attention is used to give more weight to the EPI slope information. Subsequently, multiple residual modules are used to eliminate the redundant features that interfere with the EPI slope information—in which a small stride convolution operation is used to avoid losing key EPI slope information. The experimental results revealed that the proposed algorithm outperformed the compared algorithms in terms of accuracy.

## 1. Introduction

A light field (LF) is defined as the flow of light in every 3D space. It can be represented by the two-plane parametrization as *L*(*x, y, u, v*), where the (*x, y*) plane contains the focal points of the views, and the (*u, v*) plane means image plane. *L*(*x, y, u, v*) can be viewed as an assignment of an intensity value to the ray passing through (*x, y*) and (*u, v*) [1]. LF cameras collect and record light from different directions in the scene, which can simultaneously record spatial and angular information of light rays incident at pixels of the tensor by inserting a microlens array between the main lens and image sensor [2]. The resulting plenoptic camera provides information about how the scene would look when viewed from a continuum of possible viewpoints bounded by the main lens aperture [3]. LF cameras also implicitly record the depth information, which enables many interesting applications. As a crucial step, depth estimation from images is a fundamental problem in many applications, such as in autonomous vehicle driving [4], robot navigation [5], and robot-assisted surgery [6], for which acquiring the accurate scene depth can be of great help for the related applications. Hence, improving the performance of algorithms for depth estimation contributes significantly to the field of computer vision.

Conventional depth estimation algorithms usually calculate the depth information based on the idea of stereo matching, whereas the LF images have the information of light directed from different viewpoints. The variety of presentation forms is also very rich [1]. Consequently, the rich information and the variety of presentation forms for LF images provide the possibility for improving the accuracy of the depth estimations. Currently, the depth estimation methods for LF images based on traditional algorithms are generally classified into the following categories according to the form of the input image: Multiview stereo matching algorithms based on subaperture images [7,8], algorithms based on refocused images and angle blocks [9,10,11,12], and algorithms based on epipolar plane images (EPIs) [13,14,15,16,17,18,19,20,21,22].

**Figure 1:**
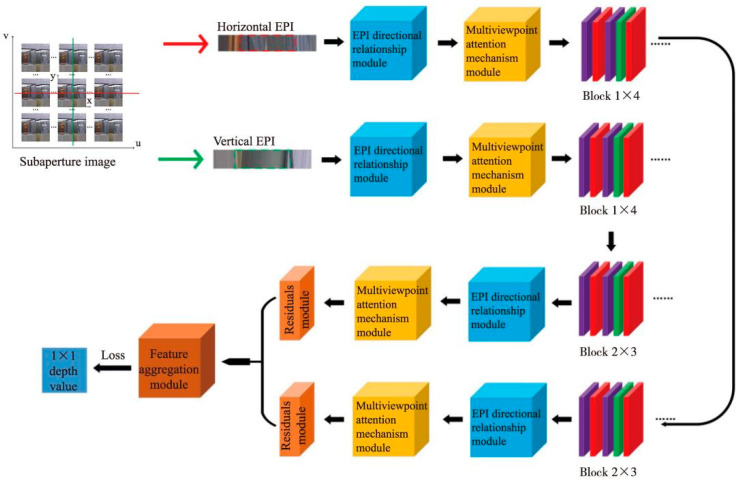
Overall framework of the proposed method. Block 1: Convolution-ReLU-Convolution-BN-ReLU with the convolution kernel size of 2 × 2 and a step size of 1. Block 2: Convolution-ReLU-Convolution-BN-ReLU with the convolution kernel size of 1 × 2 and a step size of 1.

EPI, being a visualization method unique to LF images, consists of epipolar lines which are the intersection of the epipolar plane and the camera plane. An example is shown in Figure 1. In the EPI, the adjacent line comes from the adjacent views captured by the camera. Image space disparity, defined for a pair of images captured at adjacent positions, is mapped to the displacement between two adjacent horizontal lines in an EPI. That is, the line in the EPI represents the imaging points from different views and the slope of the line indicates the disparity of the point [15]. EPIs contain both spatial and angular domain information and are more conducive to depth estimations. Consequently, the EPI LF depth estimation algorithm is able to obtain more effective depth information compared with other algorithms, and it is more beneficial to solve the occlusion problem of depth estimations. The basic idea of an EPI LF depth estimation is to find the correct EPI line and calculate the slope of the line to acquire the depth information for the corresponding pixel in the central subaperture. This method is effective for EPIs with clear oblique lines and can be applied to most scenes. However, for EPIs with occlusion regions, edge regions, and noise interference where the oblique lines are more difficult to extract [23], it is hard to obtain accurate depth information using the traditional algorithms.

EPIs, along with the rapid development of deep learning and the advances of convolutional neural networks (CNNs) in recent years, have had their applications in LF depth estimation become more and more widespread. Compared with traditional stereo matching algorithms, the LF depth estimation algorithms based on deep learning can fully extract depth information and obtain accurate LF depth information through a high-performance CNN. Generally, depending on the data presentation form, deep learning-based LF depth estimation can be divided into two kinds: subaperture image-based [7,8,24,25,26,27,28,29,30,31,32] and EPI-based methods [18,19,20,21,33,34,35]. The subaperture images contain more pixel space information than the EPI, but there are more image features, so extracting the accurate depth is the core problem of its algorithm. The EPI-based method, on the other hand, relies on the EPI slope to calculate the depth; it is more intuitive and is good at depth estimation.

In order to extract more effective slope information and improve the performance of the depth estimation, this paper proposes an EPI LF depth estimation network based on the directional relationship module and the attention mechanism. We consider two directions (horizontal and vertical) of EPIs and extract features from these two directions, respectively. After obtaining abundant EPI slope information and feature information, we combine with the two-branch structure and aggregate the final depth value of each pixel. The contributions of this paper can be summarized as:

(1) We design a directional relationship module to extract the EPI slope information. The relational model has been widely applied in the field of computer vision in areas such as target detection and semantic segmentation due to its good network performance. Mostly, the spatial pixel relationship of image features or the relationship of multichannel features are used as a cutoff and is enhanced by specific network modules to improve the network performance. Inspired by the work, the EPI directional relationship model is therefore used to extract the horizontal and vertical EPI slope information, respectively. Because more effective EPI slope information is extracted, more accurate depth results can be obtained.
(2) Considering the correlation of EPI pixels, the multiviewpoint attention module is used to process the EPI feature information. The spatial attention focuses on the correct slope at the corresponding position, the channel attention extracts the contextual information around the EPI slope, and multiple residual modules are used to eliminate other redundant features with noncorrect slope and interference information.

## 2. Related Work

In this section, we review the major works on LF depth estimation. We classify the existing methods into subaperture image-based methods and EPI-based methods.

### 2.1. LF Depth Estimation Based on Subaperture Image

The subaperture image-based method relies on multiple views of the LF images and calculates the parallax of adjacent views to obtain the depth map. In the early stage of the research, Jeon et al. [16] estimated the depth by computing matching cost volumes between the center view image and the view images that were displaced using the phase shift theorem. Wang et al. [11] introduced a depth estimation method which treated the occluded and nonoccluded regions differently to handle occlusions. Williem et al. [12] used angle entropy measurements and adaptive defocus responses to construct data costs, which are robust to occlusion.

Recently, deep learning methods have been widely used in LF depth estimations between viewpoints. Herber et al. [24] presented a convolutional neural network based on the natural LF image volumes used for shape detection, presenting for the first time the idea of using image volumes in deep learning algorithms for LF images. A pseudo-EPI-based LF image input form with four LF image volumes as network inputs was designed by Shin et al. [7] to achieve excellent depth estimation, which became the mainstream form of subaperture image input algorithms. Tsai et al. [8] designed a view selection scheme based on the attention mechanism and operated on 81 subaperture image volumes that could effectively reduce redundant features and improve the depth estimation accuracy. A multiattention mechanism network framework was designed by Chen et al. [25] to use the attention mechanism module for feature selection within and between branches of the four LF image volumes, respectively, reducing the effect of image object occlusion. Huang et al. [26] proposed a network structure—using two LF images and a central subaperture edge map as the input—to reduce occlusion interference and increase the depth estimation accuracy by reincorporating the edge information into the image volume. A highlight-resistant LF depth estimation algorithm was proposed by Wang et al. [27]. A cavity convolution was added to the network framework proposed by Shin [7] to expand the perceptual field and recover the depth information of the highlight region. Shi et al. [28] designed a multidirectional selective image method with improved Flownet [29] for parallax estimation to obtain accurate depth images. The combination of LF image volume and a single subaperture image in the form of input, and using the attention mechanism for feature aggregation was designed by Li et al. [30] to improve the network performance and enable accurate depth estimations for a wide baseline LF. Wang et al. [31] proposed a separated-light field parallax estimation and reconstruction algorithm and designed multiple network structures for separating light field subaperture images and performing feature selection and extraction, achieving very good synthesis results. Wang et al. [32] designed a mask-aware cost-based LF depth estimation algorithm based on the previous work, which integrated the matching cost by processing subaperture images with different convolution kernels. It used image edge as masks as an aid to process the mask and obtained a network model with an edge region in alignment and had strong antiocclusion performance. Even though the deep learning algorithm with subaperture images as input is able to obtain an accurate depth map, it requires complex network structures to obtain high-performance network models because the input data are too large, and the depth information is more difficult to extract directly. Furthermore, multiple subaperture images in the network will generate many redundant features that are not conducive to the network extracting important depth information, so many studies are also using other forms of LF images—such as the EPI-based network.

### 2.2. EPI-Based LF Depth Estimation

The idea of EPI-based LF depth estimation is to extract the EPI slope features using a deep learning algorithm and calculate its slope to determine the accurate depth value. Originally, Wanner et al. [15] proposed estimating the direction of the lines on EPIs based on structure tensors and then integrating the local estimation using fast denoising and global optimization. Zhang et al. [22] proposed a spinning parallelogram operator to estimate the slope of lines on EPIs by assuming the difference between the two sides of the line in the largest. After, the deep learning methods became the mainstream. Herber [33] proposed the use of CNNs to extract the depth information in the LF EPI; the network structure was relatively simple, with only a few convolutional blocks stitched together. In the same year, Herber et al. [34] proposed another U-net-based network framework for depth estimation which had improved results. However, due to the very simple structure of convolutional neural networks in the early research period and the small amount of image feature information in the EPI, the network was unable to extract deeper depth information from it and the research direction slowly developed toward the direction of using subaperture images as the input form. As deep learning research continues to evolve, many studies are using deeper and more capable network frameworks and modules to deal with LF EPIs. By designing a network framework based on two EPI blocks and postprocessing the obtained depth map using a global optimization strategy, Luo et al. [20] improved the performance of EPI LF depth estimation. Zhou et al. [18] designed a network structure with four EPI blocks, used a scale direction-aware module for feature extraction, and added refocusing cues to assist in depth estimation, and obtained better depth results. Li et al. [19] proposed a network structure based on directional relations for depth estimation, which led to better improvements in depth results. Leistner et al. [21] adopted a network structure based on EPI-shifted input, adding EPI input features and using edge masks to increase the algorithm performance and solve the wide baseline optical field depth problem. Zhou et al. [35] proposed a network structure based on hybrid inputs of subaperture images and focal stacks and proposed a new input idea to enhance the depth map effect by extracting the depth information of subaperture images through a focal stack local guidance network.

## 3. Methods

### 3.1. General Network Structure

The proposed EPI LF depth estimation method based on the directional relationship model and multiviewpoint attention mechanism will be discussed in detail in this section, as shown in Figure 1. Our network takes horizontal and vertical EPIs as its input and the output is the depth value of the corresponding pixel.

The 4D LF image is represented as *L*(*x*, *y*, *u*, *v*), where (*x*, *y*) is the spatial resolution and (*u*, *v*) is the angle resolution. By fixing two coordinates of LF images: (*y*, *v*) or (*x*, *u*), the horizontal and vertical EPIs of a pixel in the image are first obtained. They are then cropped to obtain EPI blocks containing important information of the EPI slope. We then designed a directional relationship module to extract the EPI low-level features. The high-level features of EPI slope were further extracted through the multiviewpoint attention mechanism. In addition, multiple residual modules were used to eliminate the redundant features of non-EPI slope information to accelerate the fitting speed. Finally, the depth information of the pixel was obtained after applying the feature aggregation module.

### 3.2. EPI Directional Relationship Feature Extraction Module

**Figure 2:**
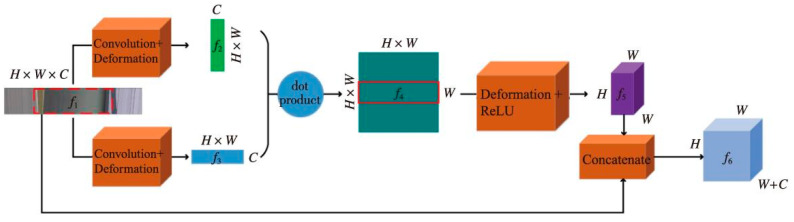
Directional relationship model.

Due to the multidirectional nature of EPI, the relational model can extract more EPI slope information from multiple directions. Therefore, this paper adopts the EPI directional relationship model for the underlying feature extraction of EPIs, which is used to obtain more EPI slope information for subsequent module processing. The EPI directional relationship module is depicted in Figure 2 in detail.

The underlying features in the EPI block *f*_1_ are first obtained by using a 1 × 1 convolutional layer after the initial EPI block *f*_1_ is input to the directional relationship module, using the correlation and compactness of the pixels in the EPI block. Then, the output features are converted into two feature forms, *f*_2_ and *f*_3_, both horizontal and vertical, as follows:

(1) $$f_{2}=Reshape_{1}[Conv(f_{1})]$$

(2) $$f_{3}=Reshape_{2}[Conv(f_{1})]$$

where *Reshape*_1_ and *Reshape*_2_ are two different feature reshapes, *Conv* is the convolution layer with a kernel size of 1 × 1, and *f*_1_ feature size is *H* × *W* × *C*; *H* is the height of the EPI block, *W* is the width of the EPI block, and *C* is the number of channels. *Reshape*_1_ reshapes *f*_1_ original *H* × *W* × *C* features to *C* × (*H* × *W*) 2D features *f*_2_. *Reshape*_2_ reshapes *f*_1_ original *H* × *W* × *C* features to (*H* × *W*) × *C* 2D features *f*_3_.

The two features are then multiplied pointwise to take full advantage of the orientation relationship in the EPI block to obtain more EPI slope information and to obtain feature *f*_4_ according to:

(3) $$f_{4}=f_{2}\cdot f_{3}$$

where · denotes the dot product and the size of feature *f*_4_ is (*H ×* *W*) × (*H* × *W*) × *W*.

After extracting the relationship feature *f*_4_ of the EPI block, the directional relationship features and the original features were combined so that the high-level features and the low-level features complemented each other. Specifically, we converted its size to a feature block *f*_5_ with the same size as the original EPI block *f*_1_ and the number of channels as the width of the original EPI block. The module output feature *f*_6_ can be defined as:

(4) $$f_{5}=Reshape_{3}(f_{4})$$

(5) $$f_{6}=Concate[f_{1},f_{5}]$$

where *Reshape*_3_ indicates the deformation operation which deforms *f*_4_ to *H* × *W* × *W*. After the *Concate* operation, we obtained the final output feature *f*_6_ whose size is equal to *H* × *W* × (*W* + *C*).

### 3.3. Multiviewpoint Attention Mechanism Feature Extraction Module

**Figure 3:**
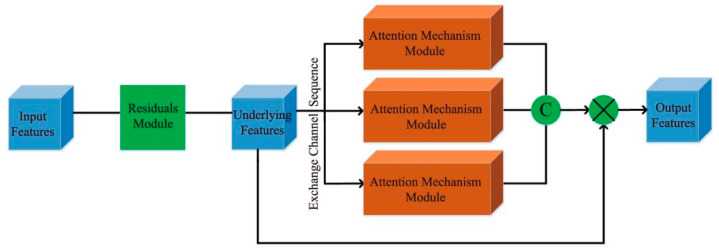
Multiviewpoint attention mechanism.

After obtaining the EPI directional relationship features, focusing and extracting the EPI slope information in the features is critical. Consequently, we further designed a multiviewpoint attention mechanism module for feature extraction after each directional relationship module, and its specific structure is illustrated in Figure 3.

In order to fully extract the correct EPI slope information in different directions, the previously obtained directional relationship features are input into three forms of the attention mechanism module. Specifically, the first channel is to perform attention mechanism processing on the horizontal EPI features to obtain the horizontal attention features. Furthermore, the second and third channels input the vertical EPI features and channel number EPI features into the attention mechanism module to obtain the vertical attention features and pixel attention features, respectively. After the three-channel part, we concatenate all the features from each channel, and make a ship connection with the original input features to obtain the final EPI features.

**Figure 4:**
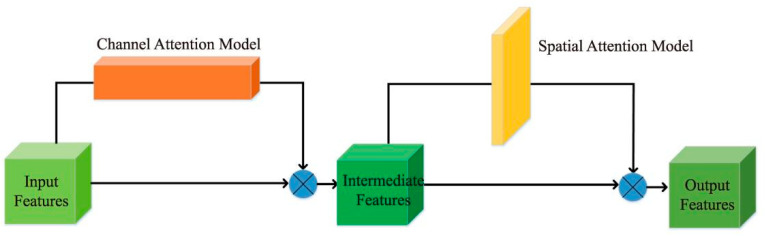
Schematic diagram of the attentional mechanism.

To extract the EPI slope information well, we adopted the attention mechanism module which combines channel and spatial features, in which features first pass through the channel attention module and focus on the feature channels that are more useful for extracting EPI slope information. Then, features pass through the spatial attention module and focus on the accurate EPI slope feature area of the EPI features, as shown in Figure 4. Furthermore, to eliminate the redundant features generated in the directional relationship module for subsequent attention mechanism modules, a residual module was designed for feature processing before each attention mechanism module.

### 3.4. Feature Aggregation Module

**Figure 5:**
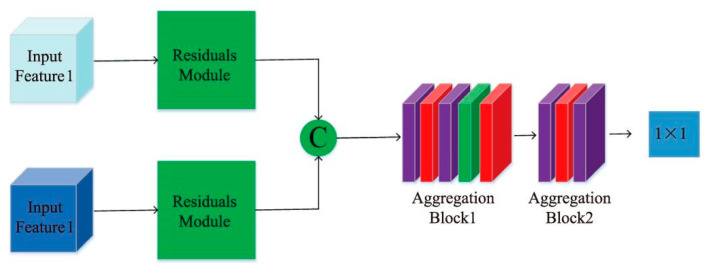
Feature aggregation module diagram.

After obtaining the EPI features completed by the attention mechanism, it is necessary to aggregate the horizontal and vertical EPI features in order to achieve the two branch features that complement each other and obtain more accurate EPI slope information. Thus, the following feature aggregation module was designed in this paper, as shown in Figure 5.

Firstly, before the feature aggregation, the redundant features that were generated in the attention mechanism need to be processed, and two EPI features need to be input to the residual module to eliminate invalid redundant features. The two branch features are then cascaded, and because the obtained EPI features are relatively small, the use of either more complex network structures or convolution operations with too-large step lengths will result in the loss of feature information. Therefore, we adopted a basic block: “Conv-ReLU-Conv-BN-ReLU” to realize feature aggregation, and most of the convolution kernels were 1 × 2 and 2 × 2 steps, with the purpose of fully extracting all EPI slope information in the aggregated EPI features and improving the accuracy of the final depth estimation.

Finally, an output of size 1 × 1 was obtained after multiple small-step convolution operations, which can be abstracted to the depth corresponding to the center of the final EPI block, thus completing the network framework construction.

## 4. Experiments

In this section, we first introduce the datasets and implementation details, then compare our method with traditional EPI methods and deep learning-based EPI methods. Finally, we discuss our failure case.

### 4.1. Datasets and Implementation Details

In this paper, we conducted experiments on a 4DHCI LF dataset to investigate our algorithm. We used 16 scenes in the “Additional” category for network training and used the “Train” and “Test” categories for model testing [36].

The initial form of the 4DHCI dataset was a subaperture image. In order to use its data for network training, it was necessary to convert the subaperture image into an EPI first. The 4D LF is denoted as *L(x, y, u, v*), where (*x,* *y*) are coordinates of pixels in the spatial domain and *(u,* *v*) are coordinates of subaperture images in the angular domain. The EPI is calculated by fixing two coordinates in different planes and changing the others. As shown in Figure 1, fixing a horizontal pixel row of constant spatial coordinate y* and constant angular coordinate v*, along the *u* axis, an array of camera views is stacked. The horizontal EPI is calculated as:

(6) $$I_{y,v}(x,u)=L(x,y^{*},u,v^{*})$$

Similarly, the vertical EPI is calculated as:

(7) $$I_{x,u}(y,u)=L(x^{*},y,u^{*},v)$$

Secondly, the size of the EPI used in this paper is 9 × 29, which is mainly because if the length of EPI image is too long, many redundant data unrelated to the EPI slope will be input into the network, affecting the efficiency of network training. If the EPI slope is too short, it will lead to the incomplete interception of the EPI slope and be unable to obtain complete and clear EPI slope data. The image input size is determined after fully considering relevant factors. Furthermore, in order to accelerate the model fitting and improve the model performance, two data augmentation algorithms are adopted for data augmentation in this paper, which are a gray scale randomization algorithm and a random Gaussian noise data algorithm. The gray scale randomization algorithm randomly changes some areas in the image to gray. In many cases, the EPI slope information is not affected by the color, which can improve the EPI slope data and training efficiency. The random Gaussian noise data enhancement is able to increase the number of training data images substantially and avoid over-fitting.

In addition, all the convolution sizes and step lengths in the framework proposed in this paper are basically chosen to be small and controlled below 2 × 2. The reason is that the EPI size is smaller than the subaperture image, and the pixel relationship contained in the EPI is more complex. In order to fully extract the EPI slope detail information, a small step convolution is taken for processing, and many Relu activation layers and batch normalization layers are used to accelerate convergence and improve training efficiency. In this paper, the method of network training was gradient descent, the batch size was set to 128, the optimizer used RMSprop, and the initial learning rate was 10^−5^. The model was trained on an NVIDIA GTX 2080Ti GPU and took about three days for training. The loss function chosen for training was the mean absolute error (MAE), which is expressed as:

(8) $$\mathrm{MAE}=\frac{1}{m}\sum_{i=1}^{m}\left|d_{gt}(i)-d_{e}(i)\right|$$

where *d_gt_* represents the real value of the *i*-th pixel, m is the total number of pixels in the depth map, and *d_e_* represents the estimated depth.

### 4.2. Quality Metrics

In order to verify the performance of the algorithm in the aspects of edge preservation, smoothness, and continuity of depth images, the BadPix (BP), mean square errors (MSE), and Q25 were used for quantitative evaluation [37]. BadPix measures the percentage of wrongly estimation pixels of which the errors exceed as:

(9) $$\mathrm{BP}(\varepsilon )=\left\{\frac{y_{i}\in m:\left|d_{gt}(i)-d_{e}(i)\right|>\varepsilon }{m}\right\}$$

Quality metrics, Q25, represents the accuracy at the 25th percentile of the disparity estimates on a given scene. Thus, it measures the maximum error on the best 25% of pixels for each algorithm. In effect, it provides an idea of the “best case accuracy” of a given algorithm.

(10) $$Q_{25}=S^{idx}\left|d_{gt}-d_{e}\right|$$

*S^idx^* is the *idx*th data sorted from largest to smallest. *|d_gt_−d_e_|* represents the absolute disparity difference between estimated depth image and ground truth. In line with the MSE, the absolute disparity difference is multiplied by 100. We set *idx* = *m* × 0.25, which represents 25 percent of the total number of pixels in the depth map.

### 4.3. Comparison to Traditional EPI Methods

Firstly, we compared our method with the mainstream LF depth estimation algorithms epi1 [14], epi2 [15], LF [16], LF_OOC [11], and CAE [17]. GT is ground truth of scenes.

**Figure 6:**
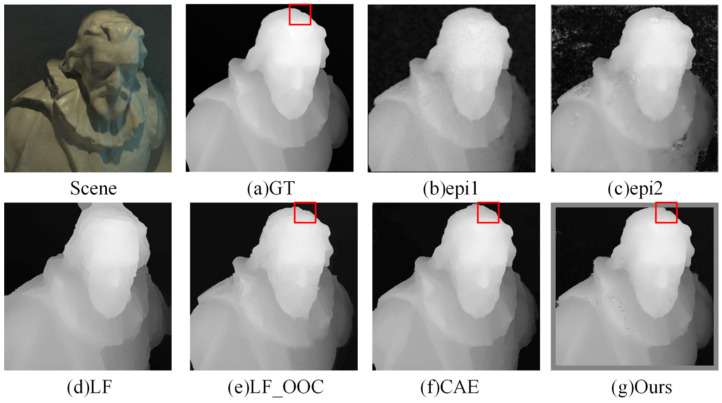
Comparison of depth results in *Cotton* scene.

**Figure 7:**
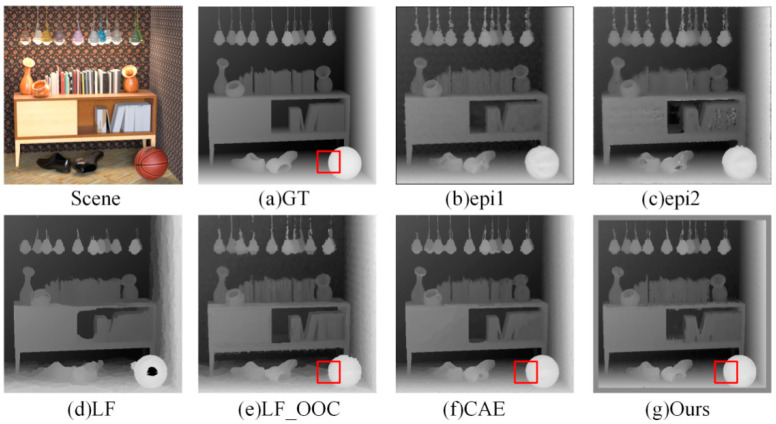
Comparison of depth results in *Sideboard* scene.

(1) Visual Comparison: Figure 6 and Figure 7 show the estimated depth maps. It can be seen that the algorithm proposed in this paper was closer to the edge of the head and chin in the *Cotton* scene—whereas the epi1 and epi2 algorithms had more noise, the LF algorithm had larger errors at the top of the head, and the LF OCC and CAE algorithms had several false bulges at the edge of the head red box compared with the true value. The comprehensive effect of the algorithm proposed in this paper is better.

Furthermore, the algorithm in this paper was also close to the true value at the edge of shoes and basketballs in the *Sideboard* scene. The edge of the red box was more accurate than in other algorithms, and the depth estimation effect was improved greatly.

(2) Quantitative Results: Quantitative comparisons of BP > 0.07 and MSE are shown in Table 1 and Table 2. Each algorithm solves the problem from a certain view and focuses on different application scenes and images according to its characteristics. It can be seen that the algorithm proposed in this paper has obvious advantages compared with the traditional LF depth estimation algorithm. In addition, the MSE index was poor in the *Sideboard* scene. Other scene indexes were better than the traditional LF image processing algorithm, and the comprehensive index was the best.

### 4.4. Comparison to Deep Learning-Based EPI Methods

For verifying the performance of the algorithm in this paper under the same class of algorithms, the experimental results are compared with the mainstream EPI deep learning LF depth estimation algorithms EPIRefocusNet [18], EPI-ORM [19], EPNosgc [20], and EPI-shift [21], and the better-performance EPI traditional LF depth estimation algorithm, SPO [22].

**Figure 8:**
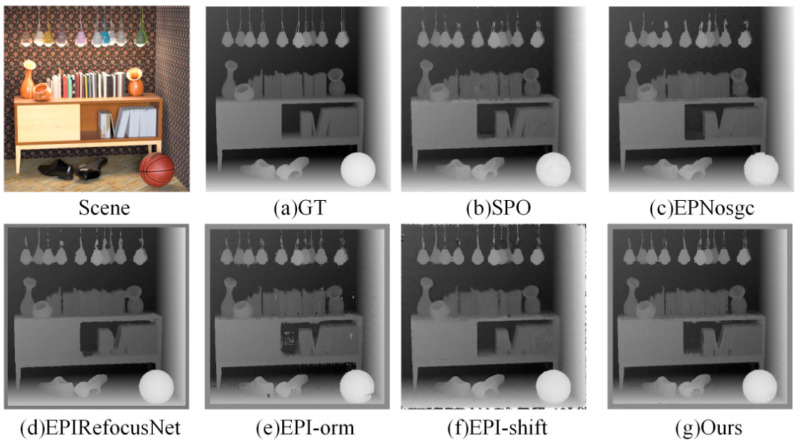
Comparison of depth results in *Sideboard* scene.

**Figure 9:**
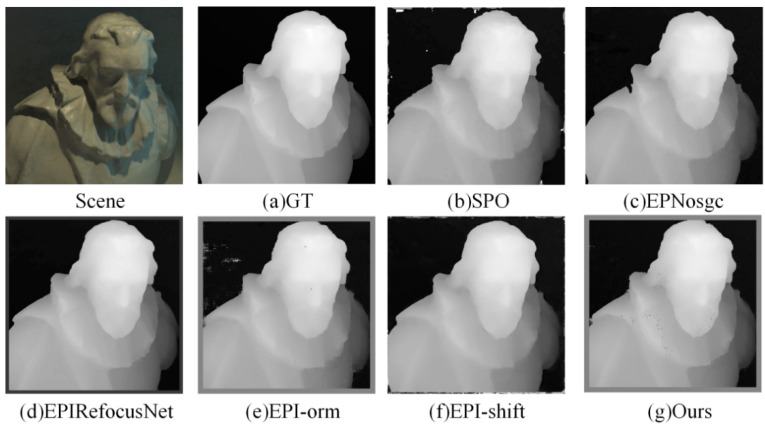
Comparison of depth results in *Cotton* scene.

**Figure 10:**
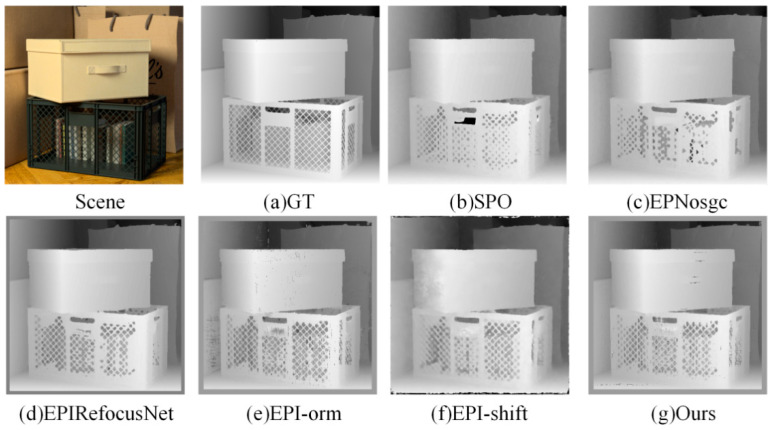
Comparison of depth results in *Boxes* scene.

**Figure 11:**
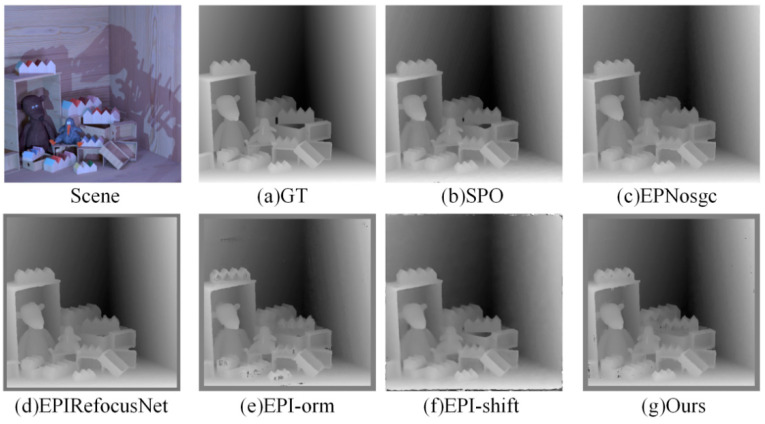
Comparison of depth results in *Dino* scene.

(1) Visual comparison on estimated depth map: Detailed experimental comparisons are shown in Figure 8, Figure 9, Figure 10 and Figure 11. It can be seen from the figures that the algorithm proposed in this paper has made a good depth prediction effect on the basketball edge in the lower right corner of the *Sideboard* scene, and the edge depth effect was very close to the true value. The edge of EPIRefocusNet and SPO algorithms had a little noise, the edge of EPNosgc and EPI-orm algorithms had error estimation, and the edge of EPI-shift algorithm had a little gap. At the chandelier region in the scene, the algorithm in this paper predicted its depth edge closer to the true value. In the region where multiple chandeliers block each other, the SPO and EPI-orm algorithms had obvious noise in the edge prediction. The EPNosgc and EPIRefocusNet algorithms had error estimations at the chandeliers, which are close to each other. The algorithm in this paper is basically accurate in predicting the edges of the chandeliers. In the *Cotton* scene, the algorithm proposed in this paper was very close to the true value in the portrait hair region prediction, and there was basically no noise in the background region—indicating that the comprehensive effect is better. In the *Boxes* scene, although the edge results of the proposed algorithm were good, there was a little noise on the box, indicating some disadvantages when compared with other algorithms. In the *Dino* scene, the proposed algorithm also had obvious effect advantages at some edges and obtained better depth results.

It can be seen from the above that the algorithm proposed in this paper is more accurate in estimating the depth detail region and can achieve good subjective results.

**Figure 12:**
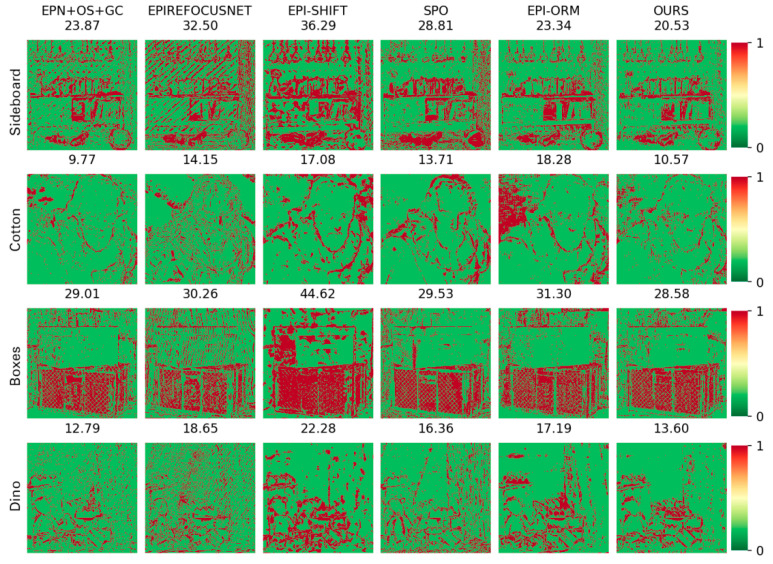
Comparison of BP > 0.03 maps.

**Figure 13:**
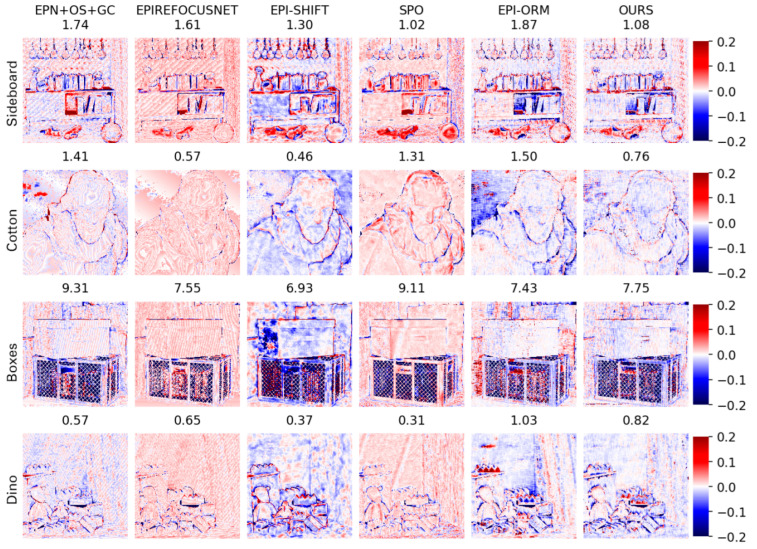
Comparison of MSE maps. Color difference reflects the change in MSE values.

**Figure 14:**
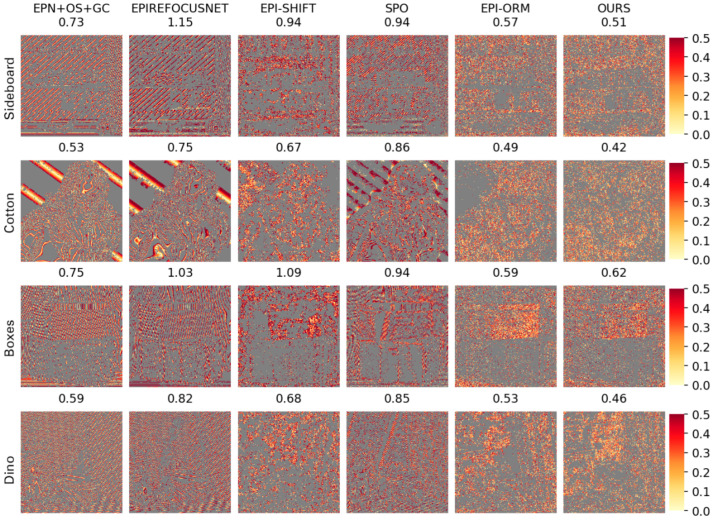
Image comparison of Q25 metrics: the absolute error of the 25% of the best pixels for each algorithm.

(2) Comparison on BP, MSE and Q25 indexes: Moreover, this paper makes three types of index correlation images based on the subjective depth map, which reflects the details of the algorithm results, as shown in Figure 12, Figure 13 and Figure 14.

BP measures the percentage of wrongly estimated pixels. It can reflect the edge preservation ability of the algorithm. As we can see in Figure 12, of the BP maps, our results had fewer false points at the edges, preserved more details, and had sharper boundaries compared to other methods. At the same time, as shown in the quantitative comparisons of BP > 0.03 in Table 3, our algorithm can always hit the optimal or suboptimal indicators. Especially for challenging scenes—e.g., the *Boxes* scene, which consists of occlusions with depth discontinuity, and the *Sideboard* scene, which had complex shape and texture—our approach always achieved the best effect. This shows that our algorithm is more advantageous for occlusion and complex scenes.

MSE reflects the smoothness of the reconstructed depth map. As shown in Figure 13 (color difference reflects the change of MSE values), although the proposed algorithm can reconstruct a relatively smooth surface and clear edges, it is easily disturbed by noise. As shown by the quantitative results in Table 4, our algorithm can achieve good results for the *Cotton* sequence containing smooth surfaces and textureless regions, but it is not dominant for noisy scenes. This is the common shortcoming of applying EPIs to the CNN-based method. The reason is that noise may lead to the false slope estimations in EPI patches. Therefore, one of the future works could introduce global constraints into our model.

Q25 visualization depicts the accuracy for those pixels that fall into the Q25, i.e., the regions with the 25% best accuracy for each algorithm. As we can see in Figure 14, our approach can reconstruct the smooth gradient in the background. Inside the object and in the background, other algorithms show stratification effects, and the gradient is not smooth (such as in the slope region of the error plot), while the algorithm proposed in this paper has almost no such phenomenon. The proposed algorithm can solve the problem that it is difficult to extract the depth of complex images in EPIs and obtains good results, which reflects the feasibility of the proposed attention mechanism to extract features.

**Figure 15:**
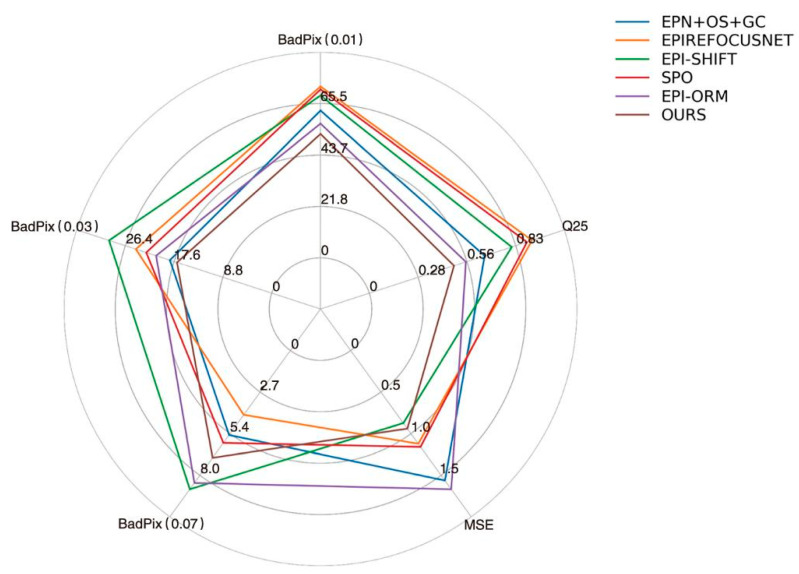
Radar chart of objective indicators.

We drew the intuitive radar chart as shown in Figure 15. It can be seen that the comprehensive performance of the proposed method in this paper is excellent, and the average effect of each scene reaches the best in BP > 0.03, BP > 0.01, and Q25 indexes. These results reflect the comprehensive performance of the algorithm proposed in this paper and proves its research value.

### 4.5. Failure Case and Discussion

**Figure 16:**
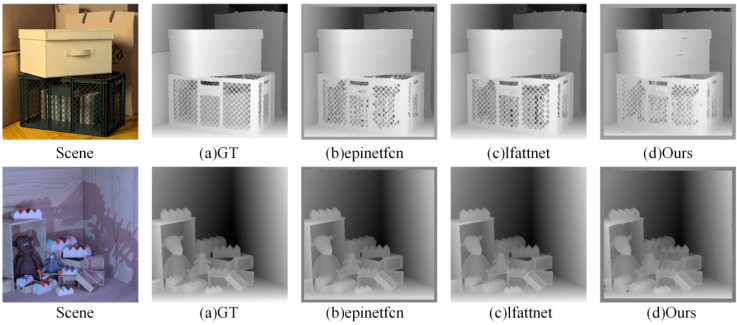
*Boxes* and *Dino* depth maps.

We further compared the proposed method with two advanced subaperture image-based methods: epinetfcn [7] and lfattnet [8]. As shown in Figure 16 and Table 5 and Table 6, our method does not achieve the best results and lfattnet obtains better performance.

Although EPI LF contains the depth information of each point in the scene, it discards many pixel features inside the subaperture image and contains less spatial information compared with other LF representation forms, which will have an impact on the accuracy of the depth estimation. Although the directional relationship model and multiviewpoint attention mechanism designed in this paper can accurately extract the EPI slope features, it is inevitable that it will lose EPI slope features due to the small number of pixels in the EPI and the occlusion of important EPI slope information. Therefore, we deduced that a multimodel LF depth estimation algorithm combining the LF EPI with other LF representation forms, such as subaperture image and focal stack images, would be designed to improve the accuracy of the depth estimation.

## 5. Conclusions

In this paper, in consideration of EPI LF’s characteristics of multidirectional relationship and pixel consistency, we proposed an EPI LF depth estimation method based on the directional relationship model and multiviewpoint attention mechanism. The EPI directional relationship model was used to establish the directional relationship of two EPIs. The EPI low-level features were extracted by using multiviewpoint attention mechanism, and the EPI slope high-level features are extracted by combining channel attention and the spatial attention mechanism. The feature redundancy was eliminated by using the residual module.

We demonstrated the effectiveness of our approach on the 4D LF benchmark. It can reconstruct the smooth surface and the region with sharp depth discontinuity. Especially, it is able to predict more accurate disparity maps in some challenging scenes such as *Boxes* and *Sideboard*. In future works, we could introduce global constraints to enhance the antinoise ability. We will also try to combine the EPI with subaperture images or focal stack images to improve the accuracy of the depth estimations.

## Figures and Tables

**Table 1:** Comparison of results for BP > 0.07.

	Bad Pixel > 0.07			
	Sideboard	Cotton	Boxes	Dino
epi1	0.1838	0.1393	0.2445	0.1035
epi2	0.1895	0.1669	0.2980	0.1567
LF	0.2199	0.0783	0.2302	0.1903
LF_OOC	0.1849	0.0622	0.2652	0.1491
CAE	0.0984	0.0337	0.1788	0.0497
Ours	**0.0887**	**0.0236**	**0.1671**	**0.0493**

Each bold indicates the best value in the corresponding column. Each underline indicates the second-best value in the corresponding column.

**Table 2:** Comparison of MSE results.

	MSE			
	Sideboard	Cotton	Boxes	Dino
epi1	2.85	2.25	8.72	1.23
epi2	4.65	4.32	10.93	2.08
LF	1.16	9.17	17.43	5.07
LF_OOC	2.30	1.07	9.85	1.14
CAE	**0.88**	1.51	8.42	**0.38**
Ours	1.08	**0.76**	**7.75**	0.82

Each bold indicates the best value in the corresponding column. Each underline indicates the second-best value in the corresponding column.

**Table 3:** Comparison of results for BP > 0.03.

	Bad Pixel > 0.03			
	Sideboard	Cotton	Boxes	Dino
EPNOSGC	23.87	**9.77**	29.01	**12.79**
EPIRefocus	32.50	14.15	30.26	18.65
EPI-shift	36.29	17.08	44.62	22.28
SPO	28.81	13.71	29.53	16.36
EPI-ORM	23.34	18.28	31.30	17.19
proposed	**20.53**	10.57	**28.58**	13.60

Each bold indicates the best value in the corresponding column. Each underline indicates the second-best value in the corresponding column.

**Table 4:** Comparison of MSE results.

	MSE			
	Sideboard	Cotton	Boxes	Dino
EPNOSGC	1.74	1.41	9.31	0.57
EPIRefocus	1.61	0.57	7.55	0.65
EPI-shift	1.30	**0.46**	**6.93**	0.37
SPO	**1.02**	1.31	9.11	**0.31**
EPI-ORM	1.87	1.50	7.43	1.03
proposed	1.08	0.76	7.75	0.82

Each bold indicates the best value in the corresponding column. Each underline indicates the second-best value in the corresponding column.

**Table 5:** Comparison of results for BP > 0.07.

	Bad Pixel > 0.07			
	Boxes	Cotton	Sideboard	Dino
epinetfcn	0.1284	0.0051	0.0480	0.0129
lfattnet	**0.1104**	**0.0027**	**0.0287**	**0.0085**
Ours	0.1671	0.0236	0.0887	0.0493

Each bold indicates the best value in the corresponding column.

**Table 6:** Comparison of MSE results.

	MSE			
	Boxes	Cotton	Sideboard	Dino
epinetfcn	6.24	0.19	0.83	0.17
lfattnet	**4.00**	**0.21**	**0.53**	**0.09**
Ours	7.75	0.76	1.08	0.82

Each bold indicates the best value in the corresponding column.
